# Gossypiboma: Spontaneous trans-urethral migration of a forgotten surgical gauze sponge 5 years post hysterectomy in grand multiparous post-menopausal woman

**DOI:** 10.1016/j.ijscr.2020.04.104

**Published:** 2020-05-19

**Authors:** J.T. Enebe, C.A. Ilo, I.J. Ofor, K.E. Chukwubuike, C.A. Omeke, N.V. Udeozor, M.N. Nwankwo

**Affiliations:** a Department of Obstetrics & Gynaecology, Enugu State University of Science and Technology College of Medicine/Teaching Hospital, Parklane, Enugu, Nigeria; b Department of Surgery, Enugu State University Teaching Hospital, College of Medicine, Nigeria; c Department of Obstetrics & Gynaecology, Enugu State University of Science and Technology Teaching Hospital, Parklane, Enugu, Nigeria; d Department of Surgery, Enugu State University Teaching Hospital, Enugu, Nigeria

**Keywords:** Gossypiboma, Gauze, Transmigration, Urinary bladder, Hysterectomy, Maternal morbidity, Case report

## Abstract

**Introduction:**

Gossypiboma denotes a mass of cotton retained in the body following surgery. Migration of gossypiboma from initial site is a rare entity and could pose some diagnostic difficulties. Migration of gauze sponge has been reported to occur in several organs of the body. There have been few reported cases but the true incidence may be much higher due to under reporting for fear of litigation.

**Presentation of case:**

We present an unusual case of a 58-year-old grand multiparous woman who had gauze retention for 5 years following a hysterectomy and presented with acute urinary symptoms. The gauze sponge transmigrated from the peritoneal cavity to the bladder and was partially extruded through the external urethral meatus. She had laparotomy for the removal of gauze sponge with good outcome.

**Discussion:**

Retained foreign body especially surgical sponges (gossypiboma) infrequently occurs and can be a source of great concern to the surgeon and patient. Foreign bodies inside the body cavities and organs can present with several non-specific clinical features that can make diagnosis difficult. Migration of surgical sponge (gauze, mops) into the urinary bladder is uncommon when compared to other abdominal and pelvic viscus. A gossypiboma in the peritoneal cavity creates a fistulous tract through the thick wall of the urinary bladder from long period of chronic inflammation as seen in the index case where the previous surgery was performed 5 years prior to onset of symptoms. Due to the non-specific presentations of gossypiboma, especially those in the bladder, several investigative modalities need to be employed to help make a prompt diagnosis. Most long-standing cases would require laparotomy due to the dense adhesions that occur around the site of the gossypiboma. Lack of appropriate diagnosis leaves the patient with recurrence of distressful symptoms and the consequent morbidities.

**Conclusion:**

Transmigration of a gauze sponge over 5 years from the peritoneal cavity into the urinary bladder and through the external urethral meatus following a hysterectomy is a rare occurrence and can present diagnostic difficulties. High index of suspicion, prompt diagnosis and management will help reduce the high morbidity that is associated with the condition as in the case reported.

## Highlights

• Gauze sponge transmigration over 5years from the peritoneal cavity to the external urethral meatus is rare but possible.
• There have been few reported cases of gossypiboma due to under reporting for fear of litigation but the true incidence may be much higher.
• Migration of gossypiboma from initial site is a rare entity and could pose some diagnostic difficulties.
• High index of suspicion and prompt diagnosis of gossypiboma is key to management to help reduce the high morbidity that is associated with the condition.
• Gossypiboma in the urinary bladder though rare but can be prevented through appropriate documentation and recounting of all surgical sponges and instruments used during surgeries.

## 1 Introduction

The term ‘gossypiboma’ denotes a mass of cotton retained in the body following surgery. The term was derived from Gossypium (cotton in Latin) and bom (place of concealment in Swahili). Migration of this gossypiboma from initial site is a rare entity and diagnosis could be difficult. Migration of the gauze sponge has been reported to occur in the duodenum, ileum, stomach, urinary bladder and lungs [[1], [2], [3], [4], [5]]. The incidence of retained surgical sponge occurs at about one per 100–300 operations [6]. The incidence maybe much higher due to under reporting for fear of litigation.

We present the case of a 58-year-old grand multiparous woman who had laparotomy with removal of gauze that has transmigrated from the peritoneal cavity to the bladder and partially extruded through the external urethral meatus. The SCARE criteria were strictly followed to produce this case report [7].

## 2 Presentation of case

A case of 58-year-old grand multiparous, 3-years postmenopausal woman, who presented with a one-month history of moderate to severe lower abdominal pain of sudden onset, continuous, crushing in character, worse with urge to defecate and micturate, relieved by intake of analgesic and not related to position. However, there was no abdominal mass or swelling, no change in bowel habit and there was no preceding trauma. There was associated urinary frequency, urgency, urge incontinence, dysuria and occasional passage of purulent fluid and blood in urine but no fever. There was no history of bleeding per vaginam, post coital bleeding and vaginal discharge; no chronic cough, bone pain but weight loss. She had total abdominal hysterectomy 5 years prior to the onset of symptoms in a private hospital due to suspected endometrial hyperplasia. However, there was no evidence of histological analysis of the tissue removed.

**Fig. 1:**
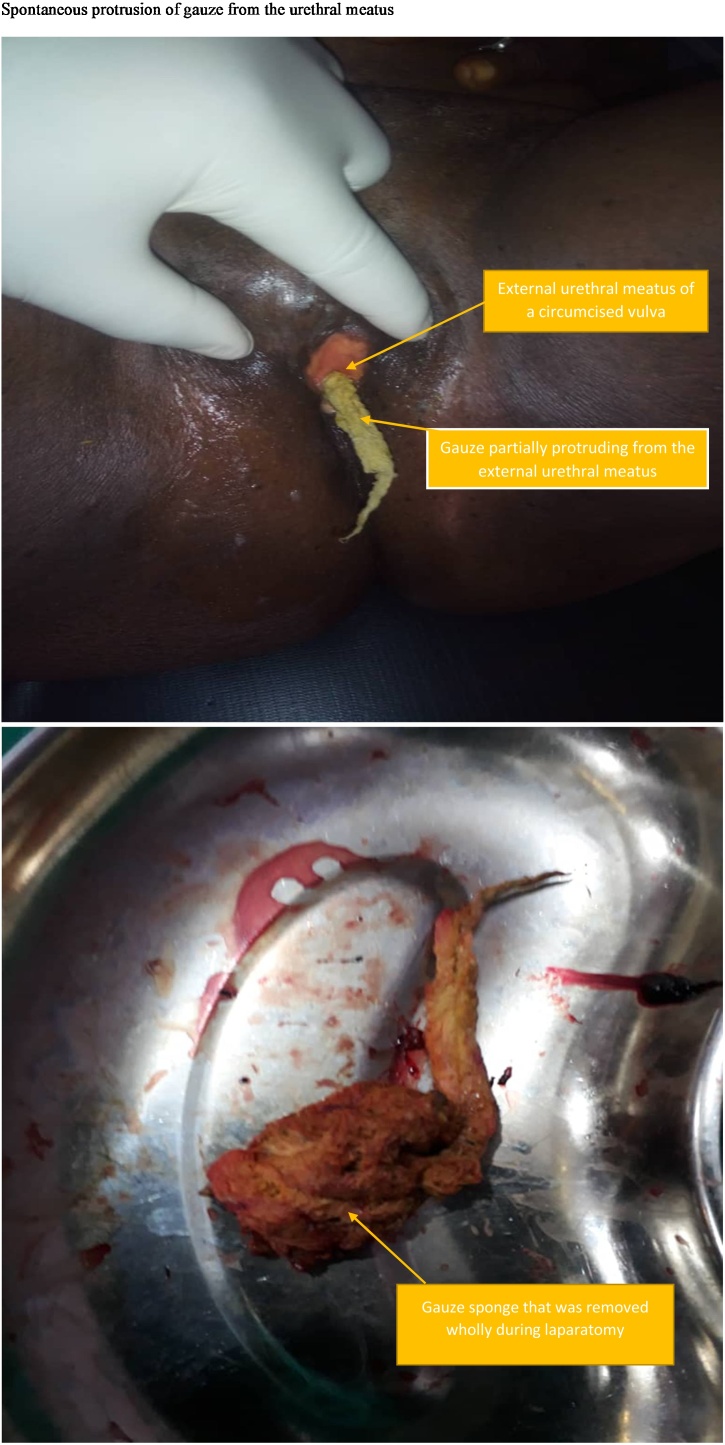
Showing gauze sponge that is partially extruded from the external urethral meatus.

On examination, her vital signs were stable, there was no palpable abdominal mass. Examination of the urogenital system revealed a gauze partially extruded from the urethral meatus with purulent discharge as shown in Fig. 1. The pelvic examination was unremarkable.

Diagnosis of cystitis secondary to foreign body in the bladder complicated by fistulous formation was made. CT urography result revealed presence of foreign body in the bladder with pelvic abscess collection. Other investigations showed normal laboratory findings. She subsequently had exploratory laparotomy for gossypiboma by a joint team of gynaecologist, urologist and general surgeons. Intraoperative findings revealed: presence of gauze shown in Fig. 1, protruding from the urethra on vaginal examination as shown in Fig. 1; dense peritoneal adhesion involving the gut, omentum and bladder, all adherent to the anterior abdominal wall; erosion of the posterior wall of the bladder with a gauze inside the bladder and purulent urine exiting from the bladder. Adhesiolysis was performed, the gauze in the bladder was removed and fistula in the posterior wall of the bladder repaired. Injury to the gut (ileum) in the course of adhesiolysis was promptly repaired.

She remained stable throughout the post-operative days and was discharged on the 13th day post operatively. She has been seen in the Gynaecology clinic on two occasions after surgery and still remained stable.

## 3 Discussion

Retained foreign body especially surgical sponges (gossypiboma) infrequently occurs and can be a source of great concern to the surgeon and patient. These foreign bodies can present with several non-specific clinical features that can make diagnosis difficult [3,5,6,8]. Migration of surgical sponges (gauze, mops) into the urinary bladder is uncommon when compared to other abdominal and pelvic viscus. Foreign bodies in the bladder can be categorized into subgroups based on how they enter the bladder [9]. These include:

Type 1: externally introduced by self for sexual gratification or as a result of psychiatric illness

Type 2: (Iatrogenic): Foreign body left at the time of major bladder surgery

Type 3 (Migratory): The foreign body usually migrates from areas/structures surrounding the bladder, commonly the uterus, rectum and vagina

Our patient had vesical migration of a gauze left in the peritoneal cavity after abdominal hysterectomy 5 years prior to onset of symptoms.

Four stages of gossypiboma migration has been proposed following experimental animal studies [10]. These are: Foreign body reaction; secondary infection; mass formation and remodeling. Gossypiboma triggers off two types of pathologic responses: Aseptic fibrinous response that creates adhesions and encapsulation; exudative response that leads to abscess formation [11].

For a gossypiboma in the peritoneal cavity to create a fistulous tract through the thick wall of the urinary bladder, there would be a long period of chronic inflammation [12,13] as seen in the index case where the previous surgery was 5 years prior to onset of symptoms. Patients with gossypiboma in the bladder may present with repeated episodes of UTI, acute urinary retention, lower abdominal pain, haematuria and occasionally protrusion of the gauze through the external urethral meatus as was seen in the index case (see Fig. 1) [3,[12], [13], [14]].

Due to the non-specific presentations of gossypiboma, especially those in the bladder, several investigative modalities need to be employed to help make a prompt diagnosis. These diagnostic modalities may involve ultrasonography, cystoscopy, CT scan, X-Ray and laparoscopy [4,8,[12], [13], [14], [15]]. Protrusion of the gauze through the external urethral meatus is diagnostic on its own as was noted in the index case (see Fig. 1). Management of this type of case may involve endoscopic removal of the gauze in piece meal through the external urethral meatus as previously documented [13]. Most cases would require laparotomy due to the dense adhesions that occur around the site of the gossypiboma especially in long standing cases as seen in the case presented [5,8,[12], [13], [14], [15]].

Gossypiboma in the urinary bladder although rare but can be prevented through appropriate documentation and recounting of all surgical sponges and instruments used during the surgery prior to closure of surgical sites/ cavities in the body.

## 4 Conclusion

Transmigration of a gauze sponge over 5 years from the peritoneal cavity into the urinary bladder and through the external urethral meatus following a hysterectomy is a rare occurrence. This condition can be associated with diagnostic difficulties. High index of suspicion, prompt diagnosis and management will help reduce the high morbidity that is associated with the condition as in the case presented.

## Declaration of Competing Interest

This is no conflict of interest to declare.

## Funding

There was no external funding received during the conduct of the research. The report was fully funded by the researchers.

## Ethical approval

Ethical approval was obtained from Ethic and Research Committee of the Enugu State University of Science and Technology Teaching Hospital, Parklane. Enugu with reference number: ESUTHP/C-MAC/RN034Nol.1/224 and dated 16th December, 2019.

## Consent

Written informed consent was obtained from the patient for publication of this case report and accompanying images. A copy of the written consent is available for review by the Editor-in-Chief of this journal on request.

## Author contribution

The contributions made by the authors are as summarised below:

**Table:** 

Authors	Contributions				
	Conception and design of the study	acquisition of data	Analysis and interpretation of data	Drafting the article or revising it critically for important intellectual content	Final approval of the version to be submitted
Enebe JTI	Yes	Yes	Yes	Yes	Yes
llo CA	Yes		Yes	Yes	Yes
Ofor IJ	Yes		Yes	Yes	Yes
Chukwu bui ke KE	Yes	Yes		Yes	Yes
Udeozor N	Yes	Yes		Yes	Yes
Omeke CA	Yes	Yes-		Yes	Yes
Nwankwo MN	Yes	Yes		Yes	Yes

## Registration of research studies

N/A.

## Guarantor

Enebe Joseph Tochukwu.

## Provenance and peer review

Not commissioned, externally peer-reviewed.
